# DSCA-PSPNet: Dynamic spatial-channel attention pyramid scene parsing network for sugarcane field segmentation in satellite imagery

**DOI:** 10.3389/fpls.2023.1324491

**Published:** 2024-01-17

**Authors:** Yujian Yuan, Lina Yang, Kan Chang, Youju Huang, Haoyan Yang, Jiale Wang

**Affiliations:** ^1^ School of Computer, Electronics, and Information, Guangxi University, Nanning, China; ^2^ Guangxi Key Laboratory of Multimedia Communications and Network Technology, School of Computer, Electronics, and Information, Guangxi University, Nanning, China; ^3^ Guangxi Institute of Remote Sensing of Natural Resources, Nanning, China

**Keywords:** deep learning, precision agriculture, remote sensing, D-scSE, PSPNet, satellite imagery, sugarcane field segmentation

## Abstract

Sugarcane plays a vital role in many global economies, and its efficient cultivation is critical for sustainable development. A central challenge in sugarcane yield prediction and cultivation management is the precise segmentation of sugarcane fields from satellite imagery. This task is complicated by numerous factors, including varying environmental conditions, scale variability, and spectral similarities between crops and non-crop elements. To address these segmentation challenges, we introduce DSCA-PSPNet, a novel deep learning model with a unique architecture that combines a modified ResNet34 backbone, the Pyramid Scene Parsing Network (PSPNet), and newly proposed Dynamic Squeeze-and-Excitation Context (D-scSE) blocks. Our model effectively adapts to discern the importance of both spatial and channel-wise information, providing superior feature representation for sugarcane fields. We have also created a comprehensive high-resolution satellite imagery dataset from Guangxi’s Fusui County, captured on December 17, 2017, which encompasses a broad spectrum of sugarcane field characteristics and environmental conditions. In comparative studies, DSCA-PSPNet outperforms other state-of-the-art models, achieving an Intersection over Union (IoU) of 87.58%, an accuracy of 92.34%, a precision of 93.80%, a recall of 93.21%, and an F1-Score of 92.38%. Application tests on an RTX 3090 GPU, with input image resolutions of 512 × 512, yielded a prediction time of 4.57ms, a parameter size of 22.57MB, GFLOPs of 11.41, and a memory size of 84.47MB. An ablation study emphasized the vital role of the D-scSE module in enhancing DSCA-PSPNet’s performance. Our contributions in dataset generation and model development open new avenues for tackling the complexities of sugarcane field segmentation, thus contributing to advances in precision agriculture. The source code and dataset will be available on the GitHub repository https://github.com/JulioYuan/DSCA-PSPNet/tree/main.

## 1 Introduction

Sugarcane, accounting for approximately 70% of the world’s sugar production (Shield, 2016) and serving as a substantial source of biofuel, is a crop with considerable economic and environmental consequences (Cardona et al., 2010; Sindhu et al., 2016). The crop’s relevance extends beyond its nutritional and energy contributions, playing an integral part in global energy security and economic stability (Moraes et al., 2015; Shield, 2016; Som-Ard et al., 2021). The escalating global population and concurrent amplification of energy demands necessitate the enhancement of sugarcane cultivation efficiency and yield optimization (Li and Yang, 2015; Som-Ard et al., 2021; Tabriz et al., 2021).

In recent years, remote sensing technology has emerged as a potent game-changer in agriculture. Its ability to provide comprehensive, accurate, and timely data is significantly altering traditional agricultural practices (Khanal et al., 2020; Weiss et al., 2020; Omia et al., 2023). This technology is particularly influential in major sugarcane-producing countries like Brazil, India, and China, where it has been instrumental in economic development and energy security (dos Santos Luciano et al., 2018; Jiang et al., 2019; Som-Ard et al., 2021). One of the key applications of remote sensing in agriculture is crop field segmentation (Sun et al., 2022; Ji et al., 2023), a process critical to various agricultural management strategies, including crop health monitoring, yield estimation, and resource allocation (Huan et al., 2021; Wang et al., 2022; Ji et al., 2023). Given its substantial downstream impacts on agricultural decision-making, achieving high accuracy levels in this operation is crucial.

To address this critical need, multiple techniques have been implemented in crop field segmentation and mapping using remote sensing data. For instance, one notable approach used a boundary-semantic-fusion deep convolution network (BSNet) to delineate farmland parcels from high-resolution satellite images, enhancing the F1 and Intersection over Union (IoU) scores (Shunying et al., 2023). An innovative open-source tool, HS-FRAG, has demonstrated its robustness by using an object-based hybrid segmentation algorithm for delineating agricultural fields, particularly in fragmented landscapes (Duvvuri and Kambhammettu, 2023). An edge detection model premised on a connectivity attention-based approach and a high-resolution structure network has been designed for farmland parcel extraction. The model introduces a post-processing method to connect disconnected boundaries, thereby enabling the generation of more refined farmland parcels (Xie et al., 2023). Similarly, a technique called the Multiple Attention Encoder-Decoder Network (MAENet) was proposed for farmland segmentation, yielding an impressive IoU score of 93.74% and a Kappa coefficient of 96.74% (Huan et al., 2021). (Bian et al., 2023) proposed CACPU-Net, linked crop type mapping with 2D semantic segmentation based on single-source and single-temporal autumn.

Sentinel-2 satellite images, achieving excellent classification accuracy on the parcel boundary. (Lu et al., 2023) proposed a multi-scale feature fusion semantic segmentation model for crop classification in high-resolution remote sensing images, providing a good reference for high-precision crop mapping and field plot extraction, while avoiding excessive data acquisition and processing.

Advancements in crop field segmentation have closely paralleled innovations in the broader arena of semantic segmentation techniques. Initially, pioneering work like the Fully Convolutional Network (FCN) introduced by (Long et al., 2015) broke new ground by replacing the conventional fully connected layer in CNNs with a convolutional layer for image segmentation. This led to alternative frameworks such as SegNet, developed by (Badrinarayanan et al., 2017), which further refined the architecture by eliminating the fully connected layer of VGGNet (Simonyan and Zisserman, 2014) and obviating the need for training during the up-sampling process. However, these early models were hampered by limitations, notably in contextual image comprehension and small object recognition, which gave rise to classification errors. Addressing these issues, the Unet model proposed by (Ronneberger et al., 2015) improved segmentation through multi-scale down-sampling and up-sampling fusion channels. To enhance global context information coherence, the Pyramid Scene Parsing Network (PSPNet) model was introduced by (Zhao et al., 2017), featuring a pyramid pooling module. Meanwhile, (Yu and Koltun, 2015) innovated by introducing dilated convolution into the traditional convolution kernel. Yet, the stacking of dilated convolutions with the same dilation rate led to information loss. The hybrid dilated convolution was proposed to address this, combining the benefits of hole convolution while reducing information loss (Wang et al., 2018). In the same vein, the DeepLab series, including V1, V2, V3, and V3+, focused on the study of dilated convolution (Chen et al., 2017a; Chen et al., 2017b). A notable advancement is the Feature Pyramid Network (FPN), which uses a top-down architecture with lateral connections to build high-level semantic feature maps at all scales (Lin et al., 2017). Recently, there has been a growing concern regarding the computational burden posed by the extensive parameters inherent in traditional semantic segmentation models. This burgeoning challenge has not only increased the demand for computational resources but has also hindered the scalability and real-time deployment of these models in resource-constrained environments. To address these limitations, the research community has directed its focus toward the development of efficient and fast semantic segmentation models (Zhang et al., 2023). One pioneering effort in this direction is the introduction of the “squeeze & excitation” mechanism in fully convolutional networks, which emphasizes channel-wise feature recalibration to adaptively emphasize informative features while suppressing less useful ones (Roy et al., 2018). This approach has been further enhanced by the Convolutional Block Attention Module (CBAM), a flexible and lightweight module that can be seamlessly integrated into any CNN architecture. CBAM refines feature maps spatially and channel-wise, ensuring that the model pays selective attention to vital regions in the input data (Woo et al., 2018). Similarly, the Squeeze-and-Excitation Networks propose a novel architectural unit that dynamically adjusts channel-wise feature responses based on the interdependencies between channels, leading to a substantial boost in model performance without considerable computational overhead (Hu et al., 2018). Collectively, these advancements reflect the broader trend in the field to optimize model efficiency without compromising accuracy, ensuring that semantic segmentation models remain applicable and effective in diverse real-world scenarios.

While semantic segmentation models have made impressive strides, their application to farmland segmentation, particularly in the case of complex crops like sugarcane, still faces a host of challenges. The quest for consistent precision in farmland segmentation, particularly for complex crops such as sugarcane, is fraught with significant challenges (Som-Ard et al., 2021). Factors including fluctuating light conditions, variations in agricultural landscapes, disparities in field sizes, and evolving crop phenology add layers of complexity to these tasks (Khanal et al., 2020; Weiss et al., 2020; Omia et al., 2023). Therefore, it is imperative to develop robust, advanced techniques that can overcome these obstacles and deliver accurate sugarcane field segmentation.

To this end, the present study introduces an innovative deep learning architecture for the segmentation of sugarcane fields, incorporating a modified ResNet34 backbone with the PSPNet and the proposed Dynamic Squeeze-and-Excitation Context (D-scSE) blocks. This proposed model efficiently addresses the complex challenges inherent in sugarcane field segmentation, outperforming traditional techniques and standard deep learning architectures. Moreover, given the importance of high-quality training data in deep learning applications, our research also contributes a novel dataset derived from high-resolution satellite imagery of Guangxi’s Fusui County in December. This dataset presents a comprehensive spectrum of environmental conditions and sugarcane field features, representing a realistic testing ground for our model and future similar applications.

The remainder of this paper is organized as follows: Section 2 details the study area, dataset characteristics, and the methodological framework underpinning our research, including the development and refinement of the DSCA-PSPNet architecture. Section 3 presents the findings from our extensive experiments, offering both qualitative and quantitative analyses of the model’s performance. In Section 4 we explore the implications of our findings, address the limitations of the current study, and outline potential avenues for future research. Finally, Section 5 synthesizes the key contributions of our work, highlighting its significance in the context of precision agriculture and its broader impact on sustainable farming practices.

In essence, the contributions of this study are threefold:

1) The study introduces an innovative deep learning model specifically engineered for sugarcane field segmentation. Utilizing a unique combination of a modified ResNet34 backbone with PSPNet and proposed novel D-scSE blocks, our model is equipped to effectively navigate through the complexities of remote sensing in agricultural landscapes.
2) The utilization and contribution of a distinctive dataset, comprised of satellite imagery from Guangxi’s Fusui County in December, stands as a valuable asset. The data capture the rich diversity of environmental conditions in the region, thus presenting a robust testing bed for our model and a valuable resource for the wider research community.
3) Our model stands apart in its performance, outperforming existing state-of-the-art segmentation techniques. Tested rigorously against established models, our approach demonstrates superior accuracy and robustness, establishing a new benchmark in sugarcane field segmentation.

## 2 Materials and methods

### 2.1 Study sites and data

#### 2.1.1 Study area

**Figure 1:**
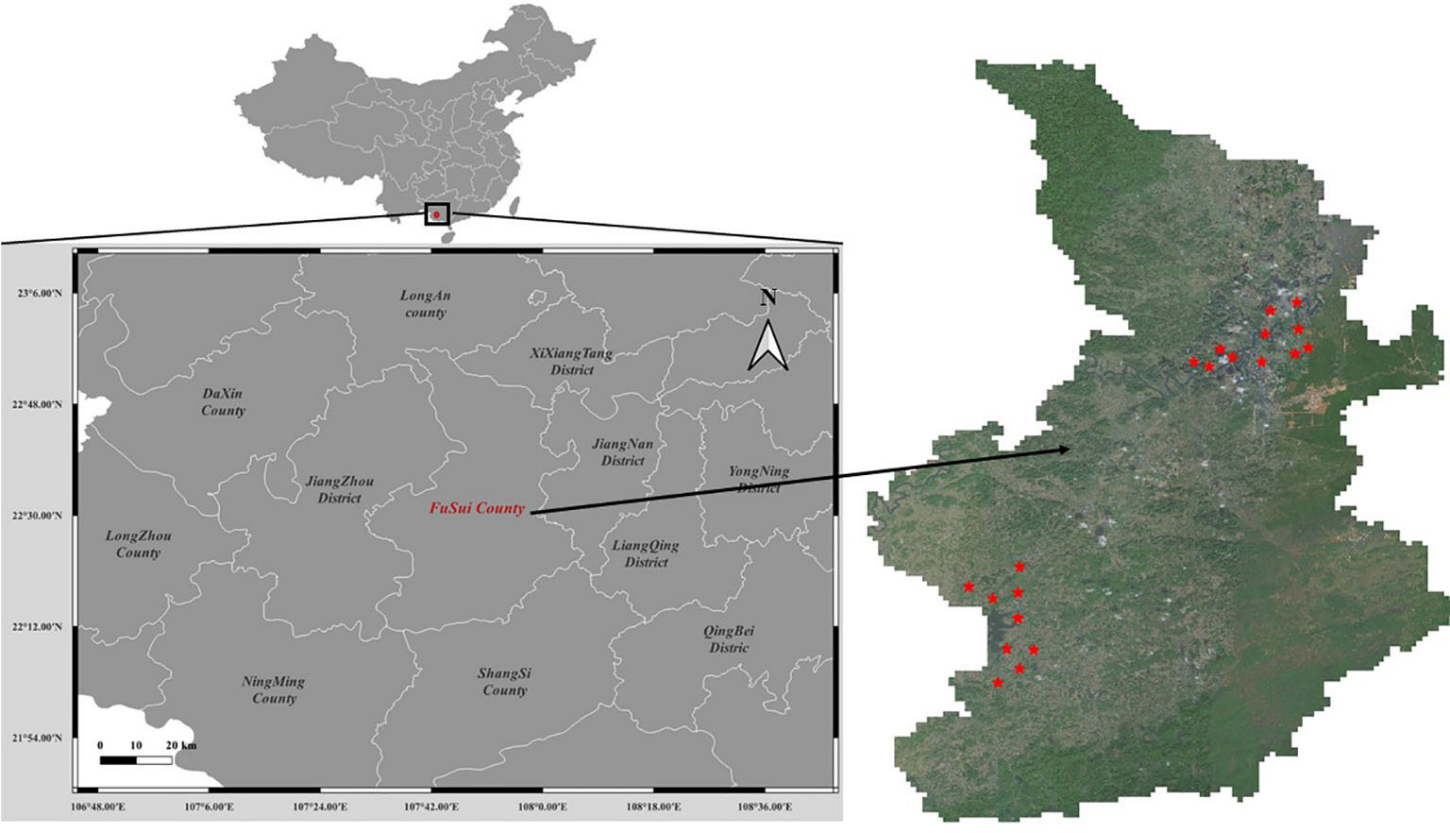
Study area.

The study area is in Fusui County (As shown in **Figure 1**), Guangxi Zhuang Autonomous Region, China, which is situated between latitudes 22°30′N and 22°47′N and longitudes 107°62′E and 107°96′E. This region is known for its extensive sugarcane production, accounting for a significant portion of the country’s sugarcane output. The climate in Fusui County is classified as a subtropical monsoon climate, characterized by hot and humid summers, mild winters, and abundant rainfall, which provides suitable conditions for sugarcane cultivation.

The landscape in this area consists of diverse terrain, including flatlands, riverbanks, and karst hills, which pose challenges for accurate sugarcane field segmentation. The complex terrain may lead to variations in the spectral signature of sugarcane fields, as well as the presence of shadows, mixed pixels, and other occlusions. Furthermore, the study area includes a range of land cover types, such as cropland, forests, water bodies, and urban areas, which can create difficulties in distinguishing sugarcane fields from other land cover types.

#### 2.1.2 Datasets

High-resolution RGB satellite images were acquired from the BJ-2 satellite on December 18th, 2017 for the study area. The images have a spatial resolution of 0.8 meters, which is suitable for identifying and segmenting individual sugarcane fields at a fine scale. Twenty remote sensing images of size 4096×4096 pixels² were selected for this study. The selected images provide a comprehensive representation of the landscape diversity and phenological stages of sugarcane fields in the region. The exact locations of these selected images are marked in **Figure 1**.

**Figure 2:**
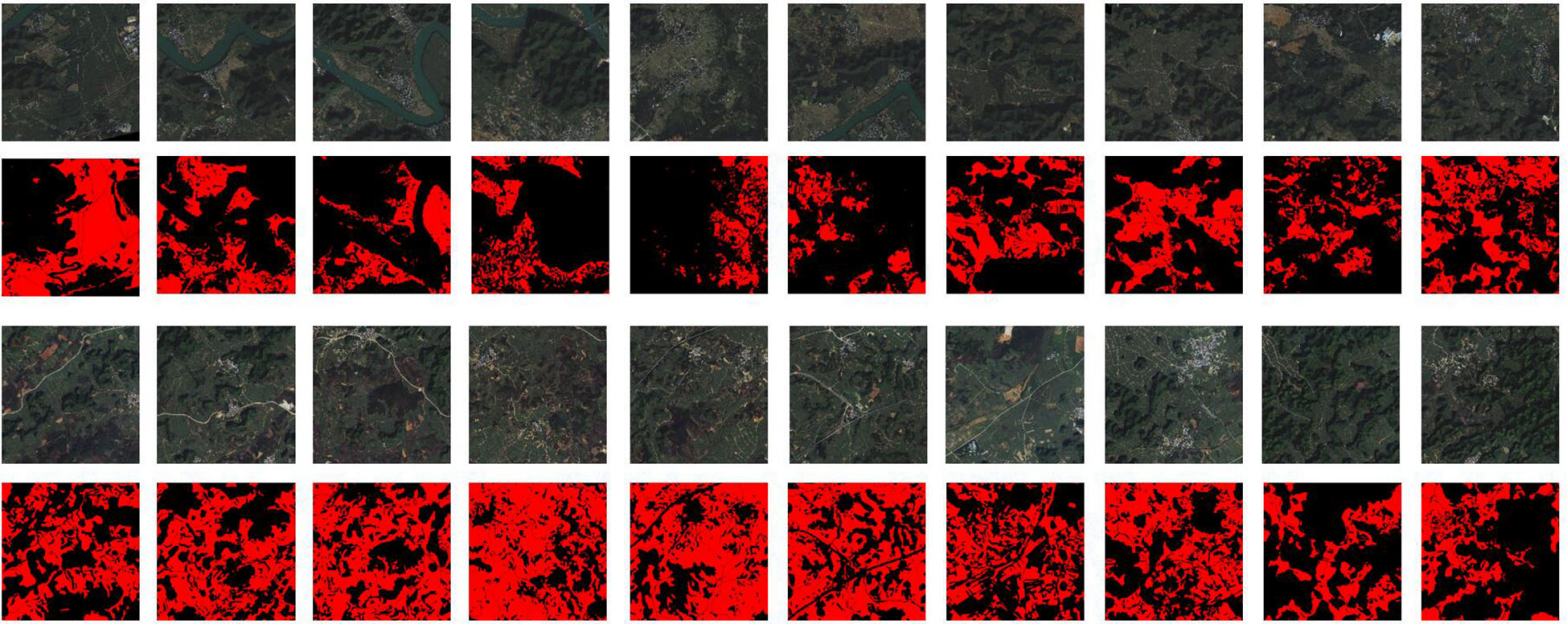
Selected images.

As shown in **Figure 2**, the images were acquired during cloud-free conditions, with minimal atmospheric haze, to ensure optimal image quality for the analysis. Additionally, the images were chosen to represent various landscape features and land cover types present in the study area, including diverse terrain, riverbanks, agricultural lands, and urban areas. This selection strategy aimed to provide a robust dataset that could effectively capture the challenges associated with accurate sugarcane field segmentation in a complex and dynamic environment.

#### 2.1.3 Data quality and preprocessing

To uphold data integrity and uniformity in this study, we embarked on a rigorous preprocessing regimen for the satellite imagery acquired from the Guangxi Institute of Natural Resources Remote Sensing (GXINRRS). These high-resolution images, captured by the BJ-2 satellite, underwent a comprehensive preprocessing protocol, including atmospheric correction, radiometric calibration, and geometric correction, using ENVI software.

The atmospheric correction stage involved adjusting specific parameters in the Fast Line-of-sight Atmospheric Analysis of Spectral Hypercubes module, accounting for aerosol optical thickness, precipitable water vapor, and atmospheric pressure. This step ensured the minimization of atmospheric distortions, thereby enhancing the representation of the ground reflectance. During the radiometric calibration phase, the sensor’s radiometric response function and the incident solar irradiance at the time of acquisition were factored in. This calibration converted the raw digital numbers in the images into standardized reflectance values, ensuring their consistent representation across different scenes. Lastly, geometric correction rectified any image distortions due to sensor geometry, Earth’s curvature, and terrain relief, utilizing the satellite’s ephemeris data, Earth’s ellipsoid and datum information, and a digital elevation model for terrain correction. This step facilitated the accurate portrayal of spatial relationships among features in the images.

#### 2.1.4 Ground truth data collection

The collection and verification of ground truth data for this study was an intricate and meticulous process involving collaboration with local agricultural experts, geography workers, and sugarcane experts. The methodology was designed to ensure accurate segmentation of sugarcane fields and robust training data for the deep learning model.

The following steps were taken in the process of ground truth data collection:

1) Image Acquisition and Preprocessing: We obtained BJ-2 satellite images of Fusui County, Guangxi, from GXINRRS and performed the preprocessing techniques mentioned in section 2.3. These images captured a diverse range of environmental conditions.
2) Expert Annotation: Agricultural and sugarcane experts and geography workers manually annotated the acquired images using ArcGIS software. They drew polygons around the sugarcane fields and delineated them by hand-drawing, utilizing their deep knowledge of local agriculture to identify these regions accurately.
3) Cross-Verification: After the initial annotation, the annotated images were cross-checked by a separate team of geography workers. They scrutinized the annotations, ensuring the masks accurately represented sugarcane fields.
4) Review and Revision: Any images that were flagged during cross-verification underwent a review and revision process. The original experts and the verification team collaborated to resolve discrepancies, resulting in a final, agreed-upon annotation.
5) Final Dataset Formation: Once all images had been annotated and verified, they were compiled into the final dataset. With its carefully validated ground truth labels, this dataset was then used for training, validating, and evaluating the proposed deep learning model.

This rigorous process, while time-consuming, was necessary to ensure the high quality and reliability of our ground truth data. This process’s collaborative and iterative nature also served to minimize human error and bias.

#### 2.1.5 Closer look at selected images and annotated masks

**Figure 3:**
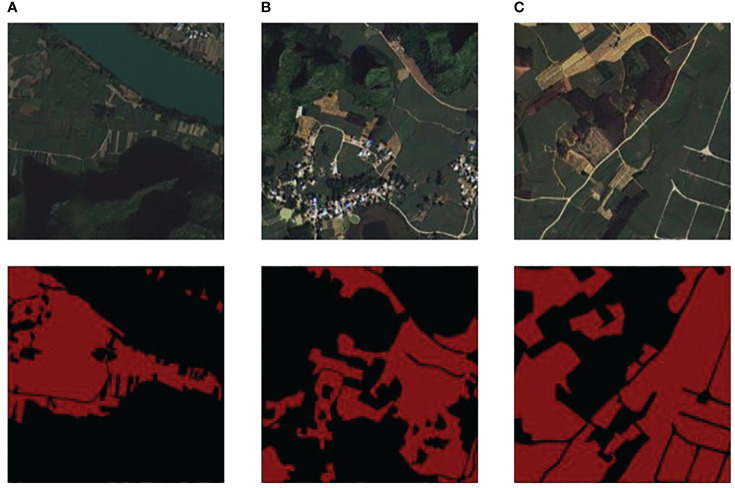
**(A)** River area and ground truth; **(B)** Resident area and label; **(C)** Farmland area and label.

To provide a comprehensive understanding of the study area and the inherent complexities it presents for sugarcane field segmentation, we examine specific images from our dataset, displayed collectively in **Figure 3**.

**Figure 3** presents a comprehensive view of three different landscapes and their corresponding segmentation maps, identified as (A), (B), and (C). In column (A), a river area is captured with features including a riverbank, karst hills, and sugarcane fields. This image presents the challenge of segmenting sugarcane fields that are intertwined with riverbanks, where water and vegetation boundaries are often indistinct. The corresponding ground truth for this area serves as the benchmark for our segmentation task. Column (B) depicts a living area with buildings, karst hills, and sugarcane fields. This scenario emphasizes the intricacy of segmenting sugarcane fields near urban structures, where the line between built and natural environments can be ambiguous. The corresponding ground truth, excluding the small roads, trees, bushes, and reaped sugarcane fields, helps in accurately distinguishing between the urban structures and natural vegetation. Lastly, column (C) portrays a farmland teeming with mixed crops and sugarcane fields. This scene highlights the difficulty of distinguishing sugarcane fields from other crop types and non-crop vegetation, which often share overlapping spectral characteristics, making the task of segmentation more complex. The corresponding ground truth excluded small roads, reaped sugarcane fields, and other non-sugarcane vegetation, which aids in deciphering the diverse crops present in the image.

Together, these images underscore the diverse challenges encountered during sugarcane field segmentation in our study area. They highlight the necessity for an advanced deep learning approach, one that is capable of grappling with these complexities and delivering precise and reliable segmentation outcomes. The source code and dataset will be available on the GitHub repository https://github.com/JulioYuan/DSCA-PSPNet/tree/main.

### 2.2 DSCA-PSPNet

#### 2.2.1 Backbone comparison

In the domain of semantic segmentation tasks, particularly for complex applications like sugarcane field segmentation from satellite images, the choice of backbone architecture substantially influences the overall model performance. For this study, we exclusively used PSPNet as the segmentation decoder, with the focus of our experimentation being on selecting the most efficient and accurate backbone. We considered six popular architectures, namely ResNet34, ResNet50 (He et al., 2016), VGG16 (Simonyan and Zisserman, 2014), EfficientNet-B5 (Tan and Le, 2019), MobileNet-V3Large (Howard et al., 2019), and ViT-B/16 (Vision Transformer) (Dosovitskiy et al., 2020), to serve as the backbone.

Experiments were carried out using the dataset and experiment settings elaborated in sections 3.3.1 and 3.3.2. The backbone architectures were compared based on metrics such as IoU, F1 scores, prediction time for a single 512x512 RGB image, number of parameters, and memory footprint. The results are concisely tabulated in **Table 1**:

**Table 1:** Metrics comparison for different backbones.

Methods	IoU	F1-Score	Prediction Time (ms)	Parameters (Million)	Memory Size (MB)
ResNet34	83.18	89.49	3.98	21.44	81.78
ResNet50	81.46	89.31	4.16	24.30	92.70
VGG16	78.85	88.09	4.98	39.34	150.09
EfficientNet-B5	81.17	89.42	7.97	28.41	108.40
MobileNet-V3Large	77.09	86.97	2.98	3.02	11.52
ViT-B/16	81.66	89.76	12.95	24.35	92.89

Based on our comprehensive evaluation, ResNet34 emerges as the most suitable backbone architecture for sugarcane field segmentation when paired with the PSPNet decoder. With a prediction time of 3.98ms, it not only facilitates real-time inference but also operates with a manageable number of parameters (21.44M), thereby making it amenable to deployment in resource-constrained environments. Furthermore, its memory requirement is 81.78 MB, while maintaining high IoU and F1 scores, indicative of its accuracy and reliability. Consequently, for the specialized task of semantic segmentation in agricultural settings, the balanced and robust performance of ResNet34 substantiates its selection as the backbone architecture.

#### 2.2.2 Modified ResNet34 backbone

ResNet (He et al., 2016) is a family of deep residual networks that effectively addresses the degradation problem in deep neural networks by introducing residual connections. In this study, we utilize the ResNet34 architecture as our model’s backbone, with specific modifications tailored to the task of agricultural crop field segmentation.

**Figure 4:**
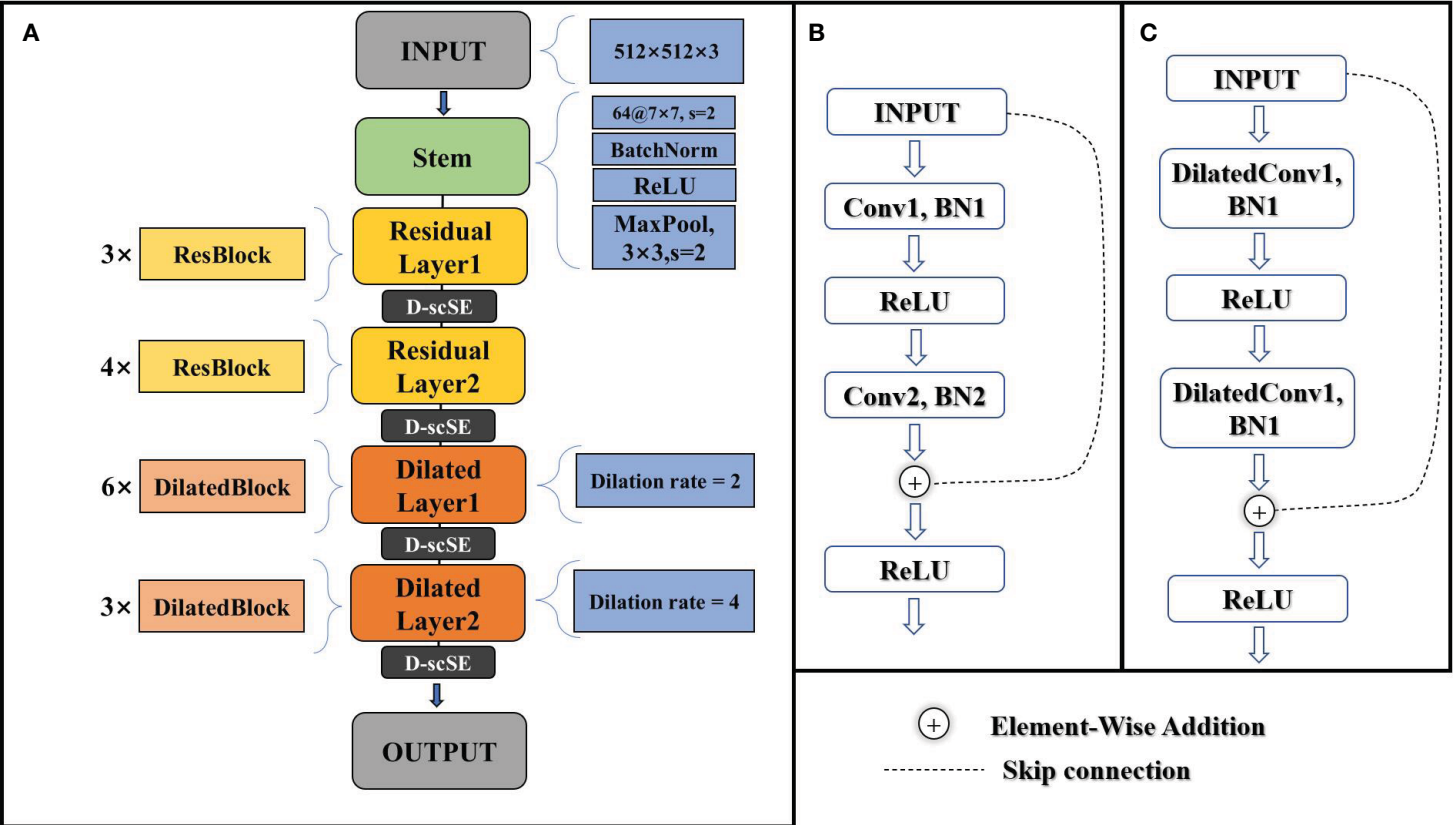
**(A)** schematic of the modified ResNet34 architecture. **(B)** schematic of the standard residual block. **(C)** schematic of the dilated residual block.

As illustrated in **Figure 4A**, the modified ResNet34 backbone consists of several components. It begins with an input layer, followed by a stem composed of a convolutional layer, batch normalization, and a ReLU activation function. The stem is succeeded by two residual layers, each containing a series of standard residual blocks, as depicted in **Figure 4B**. These residual layers capture local features in the input images.

The latter part of the backbone includes two dilated layers, with dilated blocks that incorporate dilated convolutions (Yu and Koltun, 2015), as shown in **Figure 4C**. The dilated blocks allow for a larger receptive field without increasing the number of parameters or computational complexity. The final output layer generates high-level feature maps for the input images.

The modified ResNet34 backbone integrates the advanced D-scSE attention mechanism after each residual layer (layer1, layer2, layer3, and layer4), enhancing channel and spatial dependencies and refining feature representation. The inclusion of the D-scSE mechanism improves the model’s ability to capture essential contextual information, leading to more precise segmentation results. A detailed examination of the D-scSE mechanism’s design and its role in augmenting the modified ResNet34 backbone will be provided in section 3.3.

The architecture’s larger receptive field, achieved by incorporating dilated convolutions in the later stages, is especially beneficial for capturing contextual information in high-resolution agricultural imagery with objects spanning various spatial scales. By incorporating these modifications, the backbone design provides an effective foundation for the decoder to generate accurate agricultural crop field segmentation maps, addressing the specific challenges of the task and leveraging the power of residual networks, dilated convolutions, and the D-scSE mechanism.

#### 2.2.3 D-scSE block

The D-scSE mechanism, where “D” stands for “Dynamic,” is an advanced attention mechanism inspired by the original scSE (Roy et al., 2018). While the original scSE effectively encodes channel and spatial dependencies, it doesn’t account for the varying importance of these aspects across different input data or stages of network depth. The importance of spatial and channel-wise features may dynamically vary based on the contextual information in the scene, or the intricacy of the features being learned at different network layers. This limitation could potentially restrict the learning capacity and performance of the original scSE.

To overcome this, the D-scSE mechanism introduces dynamic weights, providing a more adaptive balancing between the significance of spatial and channel-wise information. These weights are learned during the training process, offering the flexibility to modulate the degree of attention applied to the spatial and channel dimensions based on the input’s inherent characteristics.

In this section, we will delve into the specifics of the D-scSE’s design, its components, and the way it refines feature representation. We’ll discuss how this dynamic weighting scheme leads to enhanced feature learning and contributes to the overall efficacy of our proposed model architecture.

**Figure 5:**
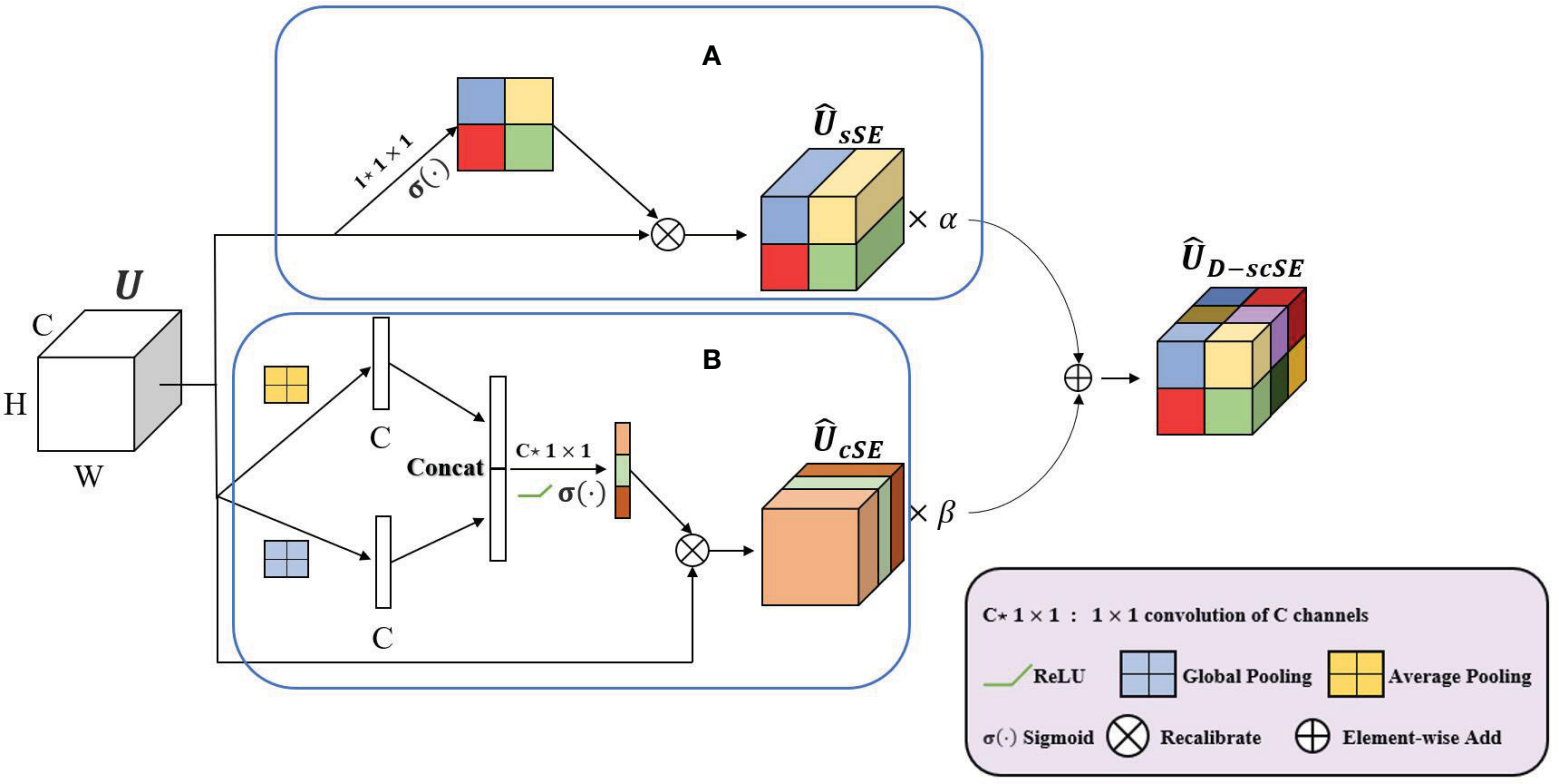
A schematic representation of the D-scSE mechanism. **(A)** sSE module and **(B)** cSE module.

1) Channel Squeeze and Spatial Excitation Block (sSE): This block focuses on spatial information, as shown in **Figure 5A**. The input feature map *U ∈ R* ^*C*×*H*×*W*^ is first channel-wise squeezed using a.

1 × 1 convolution (Equation 1):


(1) $$\text{U}=\left[u^{1,1},u^{1,2},\ldots ,u^{i,j},\ldots ,u^{H,W}\right]\cdot \text{ with }u^{i,j}\in {\text{R}}^{\text{C}\times 1\times 1}$$

The spatial squeeze operation computes the output matrix $k\in R^{H\times W}$ (Equation 2):


(2) $$\text{k}={\text{W}}_{\text{k}}⋆\text{U}$$

where $W_{k}\in R^{C\times 1\times 1}$ and ⋆ denotes the convolution operation. The spatial information weight is added to the feature map *U* by applying the sigmoid activation function (·) 

to each element in *k* (Equation 3):


(3) $${\hat{U}}_{sSE}=F_{\text{scale}}\left(U,k\right)=\left[\sigma \left({\text{k}}_{1,1}\right){\text{u}}^{1,1},\ldots ,\sigma \left(k_{i,j}\right)u^{i,j},\ldots ,\sigma \left(k_{H,W}\right)u^{H,W}\right]$$

2) Spatial Squeeze and Channel Excitation Block (cSE): This block focuses on channel-wise dependencies, as shown in **Figure 5B**. The input feature map *U* is first spatially squeezed using global average pooling and global max pooling (concatenated) before passing them through the convolutional layers (Equation 4):


(4) $$\text{x}=\text{ Concat }\left(\frac{1}{\text{H}\times \text{W}}\sum_{\text{i}}^{\text{H}}\sum_{\text{j}}^{\text{W}}\text{U}\left(:,\text{i},\text{j}\right),\underset{\text{i}=1,\ldots ,\text{H};\text{j}=1,\ldots ,\text{W}}{\text{max}}\text{U}\left(:,\text{i},\text{j}\right)\right)$$

To discern the dependency information between channels, a single fully connected layer is employed, with weights $\;W\in R^{C\times 2C}$ . Activation of this layer is achieved through the application of the ReLU function (·) and the sigmoid function *σ*(·) (Equation 5):


(5) s=σ(Wδ(x))

The final output is obtained by re-scaling the transformation *U* (Equation 6):


(6) $${\hat{U}}_{cSE}={\text{F}}_{\text{scale}}(\text{U},\text{s})=\text{s}⋆\text{U}$$

We introduce dynamic weighting to balance the contributions of the sSE and cSE branches to the final output. The outputs of the sSE and cSE branches are combined (Equation 7):


(7) $${\hat{U}}_{D-scSE}=\alpha {\hat{U}}_{sSE}+\beta {\hat{U}}_{cSE}$$

where α and β are learnable parameters initialized by sampling from a uniform distribution $U\left(-\sqrt{6/n},\sqrt{6/n}\right)$ where *n* is the number of input units in the weight tensor. These dynamic weights are updated during the training process, allowing the D-scSE module to adaptively balance the importance of spatial and channel information based on the input data.

D-scSE module enhances the original scSE mechanism by integrating dynamic weighting and diversified pooling strategies, as shown in **Figure 5**. With the sSE branch concentrating on spatial information and the cSE branch addressing channel-wise dependencies, the module effectively recalibrates both dimensions of the feature map. By employing learnable weights, the D-scSE module adeptly balances spatial and channel information, ultimately delivering a robust feature extraction mechanism for the segmentation task.

#### 2.2.4 Pyramid scene parsing network decoder

In our proposed architecture, we utilize the PSPNet decoder, originally introduced by (Zhao et al., 2017), to generate high-quality segmentation results. The decoder effectively captures contextual information from the output feature map of the encoder by leveraging pyramid parsing and fusing multi-scale features. Additionally, the decoder is integrated with the D-scSE mechanism to further refine the feature representation.

The decoder comprises the following components:

1) Pyramid Pooling Module: This module is designed to extract contextual information from the input feature map by applying multiple pooling operations with varying kernel sizes. This approach enables the capture of both local and global context at different scales. The pyramid pooling module consists of four parallel branches, each employing an average pooling layer with a unique kernel size. Subsequently, a 1x1 convolution is used to reduce the number of channels to a predefined number (e.g., C/4). The resulting feature maps are then upsampled to their original spatial dimensions using bilinear interpolation.
2) Feature Concatenation: The upsampled feature maps originating from the pyramid pooling module are concatenated with the initial input feature map, facilitating the fusion of multi-scale contextual information.
3) D-scSE Mechanism: As detailed in Section 3.3, the D-scSE mechanism is incorporated following the feature concatenation step to adaptively recalibrate the spatial and channel-wise information. The inclusion of the D-scSE mechanism within the decoder further refines the feature representation, enabling the model to better manage varying object scales and shapes.
4) Final Convolution Layers: After implementing the D-scSE mechanism, the feature map is processed through a series of convolutional layers to generate the ultimate output segmentation map. This typically consists of one or more 3x3 convolutions, followed by a 1x1 convolution to project the feature map onto the desired number of output classes. The final segmentation map is then upsampled to match the original input image size using bilinear interpolation.

**Figure 6:**
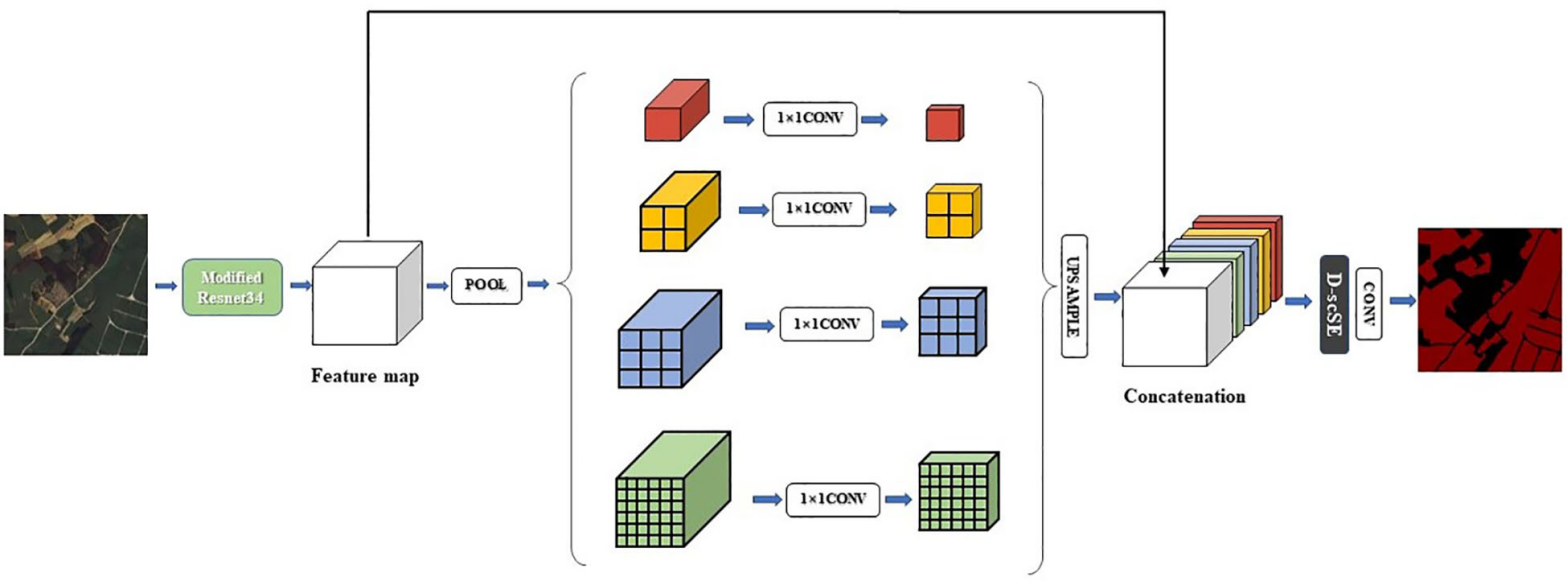
Structure of DSCA-PSPNet.

By integrating the PSPNet decoder with the D-scSE mechanism, DSCA-PSPNet (as shown in **Figure 6**) effectively captures and exploits multi-scale contextual information, thereby enhancing segmentation performance. This decoder design contributes to the generation of more accurate and finer-grained segmentation maps, ultimately improving the overall efficacy of the architecture.

### 2.3 Experiments

#### 2.3.1 Data preparation and augmentation

To create a diverse and representative dataset for model validation, twenty remote sensing images of size 4096x4096 pixels² were selected from the remote sensing images of Fusui County in Guangxi Zhuang Autonomous Area. The locations of the data samples were selected based on the presence of different land features, such as river areas, farmland areas, and living areas.

Each of the twenty original 4096×4096 images was cropped into sixty-four 512×512 images, resulting in a total of 1280 images. This cropping is a standard practice in semantic segmentation tasks, especially when handling high-resolution imagery, to manage GPU memory constraints and optimize computational efficiency. While this approach divides larger sugarcane plots into smaller segments, it does not significantly impact the segmentation task. Our model is designed to accurately classify each pixel within these segments, ensuring effective and reliable segmentation across the cropped images. To ensure a balanced dataset for model training and evaluation, 70% of the cropped images from each original image were allocated to the training set, 15% were assigned to the validation set, and the remaining 15% were assigned to the test set. This partitioning strategy ensured that the training, validation, and test sets contained a diverse range of features and challenges associated with sugarcane field segmentation.

Data augmentation techniques were applied to increase the diversity of the training dataset, making the model more robust and capable of handling real-world scenarios. The augmentation techniques applied to the dataset include rotation, horizontal and vertical flipping, random scaling, random brightness and contrast adjustment, addition of Gaussian noise, Gaussian blur, and hue, saturation, and value adjustment. These augmentations were performed using the Albumentations Python library. For each original training sample, 5 augmented samples were generated by applying all the aforementioned augmentation techniques simultaneously. This resulted in an augmented dataset of 4480 samples. Hence, the distribution of samples among the training, validation, and test sets as shown in **Table 2**.

**Table 2:** Sample distribution across training, validation, and test sets .

Dataset	Original Images	Augmented Images	Total Images
Training set	896	4480	5376
Validation set	192	0	192
Test set	192	0	192

#### 2.3.2 Experimental design

The experiments conducted in this study, which encompassed the training, validation, and testing of the proposed model, were performed on a system equipped with the Windows 10 System. The experimental runtime environment was set up using Anaconda3, Python 3.10.5, CUDA 11.7, and OpenCV 4.6. The hardware used for the experiments included 64 GB RAM, Intel (R) Core i9-10980XE@3.00GHz processor, and a NVIDIA RTX 3090 GPU. Pytorch was chosen as the deep learning framework for implementing the proposed model.

The purpose of the experiments in this study was to verify the effectiveness of the proposed model, in the recognition of sugarcane field. The fed images were 512×512. The AdamW optimizer, an improvement over traditional Adam by decoupling weight decay from the optimization steps, was utilized to prevent overfitting and achieve faster convergence. The learning rate was controlled using a cyclical learning rate strategy. The base learning rate was set to 0.0001, and it cyclically varied between this value and a maximum of 0.001, facilitating optimal convergence. Other hyperparameters included an epoch count of 100 and a training batch size of 16.

#### 2.3.3 Evaluation metrics

The accuracy, precision, IoU, F1 score, and Recall were calculated (Equations 8–12) and used as the accuracy evaluation indexes of the experimental results in this study, that is,


(8) $$\text{IoU}=\frac{\text{TP}}{\text{TP}+\text{FP}+\text{FN}}$$

(9) $$\text{Accuracy}=\frac{\text{TP}+\text{TN}}{\text{TP}+\text{TN}+\text{FP}+\text{FN}}$$

(10) $$\text{Precision}=\frac{\text{TP}}{\text{TP}+\text{FP}}$$

(11) $$\text{Recall}=\frac{\text{TP}}{\text{TP}+\text{FN}}$$

(12) $$\text{F}1=2\times \frac{\text{Precision}\times \text{Recall}}{\text{Precision}+\text{Recall}}$$

where TP denotes positive samples correctly classified by the model, FN denotes positive samples incorrectly classified by the model, FP denotes negative samples incorrectly classified by the model, TN denotes negative samples correctly classified by the model.

Accuracy is depicted as the fraction of pixels that were accurately predicted, in contrast to the total sum of pixels. Precision constitutes an evaluative metric to gauge the accuracy of predictions within a specific category. IoU is a statistical measure that identifies the degree of overlap between the predicted and the original annotated regions within an image. The F1 score is the harmonic mean of precision and recall, serving as a balanced estimator of the classifier’s performance. In addition, recall, also known as sensitivity or true positive rate, quantifies the proportion of actual positives that are correctly classified. It is an integral part of the evaluation schema, examining the classifier’s proficiency in identifying all the pertinent instances within the dataset.

## 3 Results

### 3.1 Contrast experiments

In this revised section, we will first delve into the qualitative analysis through the visual examination of segmentation results and subsequently provide a quantitative examination through the rigorous metric evaluations. Our objective remains to present a coherent and comprehensive comparison of the proposed DSCA-PSPNet with the benchmark models: Unet, DeepLabV3+, FPN, and PSPnet.

**Figure 7:**
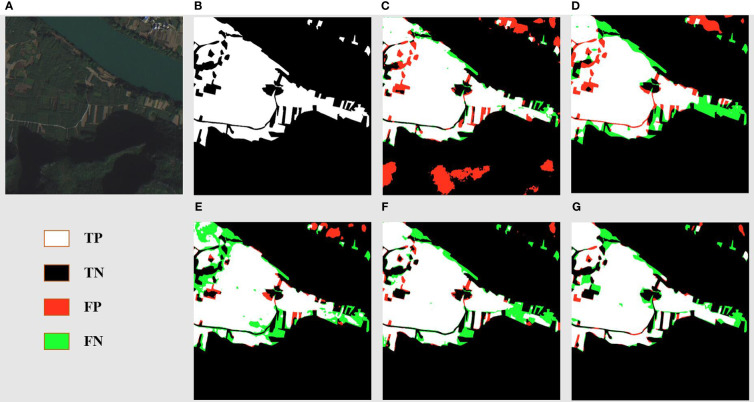
River area prediction results of models. **(A)** Original Images. **(B)** Ground Truths. **(C)** Unet. **(D)** Deeplabv3+. **(E)** FPN. **(F)** PSPnet. **(G)** DSCA-PSPNet).

**Figure 7** displays the segmentation results for a landscape marked by reaped land, sugarcane fields, and river banks. The original images (**Figure 7A**) elucidate a complex environment where sugarcane fields fringe the river banks, interspersed with fragments of reaped land. The ground truth (**Figure 7B**) meticulously captures the distinct boundaries between these zones. DSCA-PSPNet (**Figure 7G**) demonstrates a remarkable alignment with the ground truth, adeptly segment the sugarcane fields from adjacent reaped land and preserving the nuanced contours of the karst hills. In contrast, Unet (**Figure 7C**) falsely recognizes the karst hills green vegetation as the sugarcane field, blurring the transition between karst hills and sugarcane fields. Deeplabv3+ (**Figure 7D**) provides a robust segmentation of sugarcane fields, but the delineation of reaped land seems slightly generalized. FPN (**Figure 7E**) exhibits a slightly better results but the miss segmentations are still existing. PSPnet (**Figure 7F**) offers balanced performance, although minor miss segmentations are evident, especially in regions where sugarcane fields are situated in the narrow land between river and hills. Collectively, the comparative analysis underscores DSCA-PSPNet’s superior capability in effectively segmenting complex riverine landscapes.

**Figure 8:**
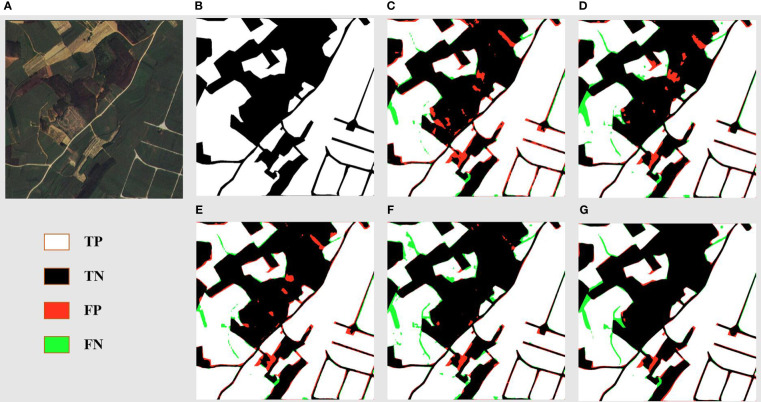
Farmland area prediction results of models. **(A)** Original Images. **(B)** Ground Truths. **(C)** Unet. **(D)** Deeplabv3+. **(E)** FPN. **(F)** PSPnet. **(G)** DSCA-PSPNet).

**Figure 8** offers a detailed segmentation analysis of a landscape primarily characterized by sugarcane fields, reaped land, other vegetation, and minor road networks. The ground truth (**Figure 8B**) accurately maps out these features, showcasing the stark boundaries between cultivated sugarcane fields, reaped areas, other vegetation, and the intricate web of roads. DSCA-PSPNet (**Figure 8G**) mirrors this ground truth with impressive precision, successfully delineating the sugarcane fields from reaped patches and capturing the delicate intricacies of the minor roads and other vegetation patches. In comparison, Unet (**Figure 8C**) occasionally confuses the reaped land with lighter patches of sugarcane fields, leading to minor segmentation inconsistencies. Deeplabv3+ (**Figure 8D**) effectively segments the larger sugarcane plots but sometimes overlooks the subtle distinction between reaped land and lighter sugarcane fields. FPN (**Figure 8E**) provides a commendable segmentation but faces challenges in accurately mapping the other vegetations. PSPnet (**Figure 8F**) produces a balanced segmentation but has minor discrepancies in areas where roads intersect with reaped land and other vegetations. Collectively, the comparative evaluation emphasizes DSCA-PSPNet’s robust capability in accurately segmenting a multifaceted farmland environment, highlighting its promise for precision agriculture applications.

**Figure 9:**
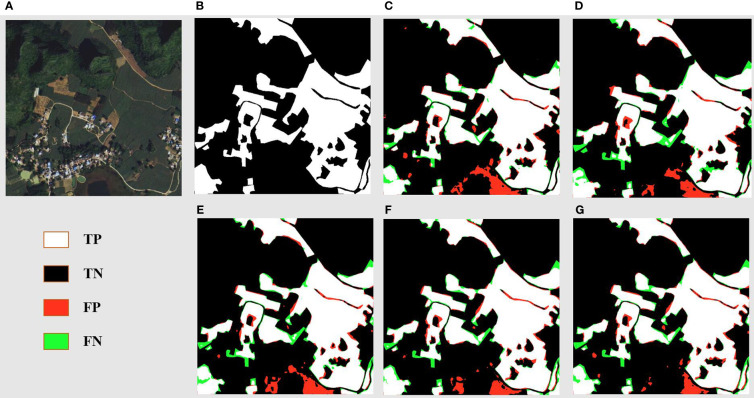
Resident and farmland area prediction results of models. **(A)** Original Images. **(B)** Ground Truths. **(C)** Unet. **(D)** Deeplabv3+. **(E)** FPN. **(F)** PSPnet. **(G)** DSCA-PSPNet).

**Figure 9** delves into the segmentation of a landscape in the residential zones with sprawling farmland areas. DSCA-PSPNet (**Figure 9G**) emerges as a standout, replicating the ground truth with exceptional accuracy. It captures the structured layout of residential zones and small roads, and has the minimal miss segmentations in water pond area. In contrast, Unet (**Figure 9C**) exhibits challenges in accurately segmenting the water pond region. Deeplabv3+ (**Figure 9D**) adeptly identifies the larger residential blocks but seems to slightly oversimplify the segmentation of smaller farmland patches situated between residential clusters. FPN (**Figure 9E**) offers a respectable segmentation but shows major miss segmentation in water pond region too. PSPnet (**Figure 9F**) provides a consistent segmentation but faces minor deviations in areas where dense vegetation in farmlands is proximal to residential zones. In summation, the analysis underscores DSCA-PSPNet’s superior ability in segment the residential and farmland landscapes, showing its power in mixed-use land segmentation tasks.

To complement the qualitative insights underscoring the enhanced performance of DSCA-PSPNet, we shall now transition to a quantitative analysis that empirically substantiates these observations.

Evidently, DSCA-PSPNet stands out across all evaluation metrics, reinforcing its potency as affirmed by the visual outcomes. Specifically, DSCA-PSPNet records an IoU of 87.58%, indicative of its exceptional overlap prediction ability, leading the second-best performer, PSPnet-resnet34, by a significant margin of 4.4%. Its accuracy score of 92.34% is the highest among all models, reflecting the model’s impressive capability in classifying each pixel correctly. In terms of precision, DSCA-PSPNet’s score of 93.8% further cements its supremacy, signaling its strength in minimizing false positives, outperforming the runner-up, FPN-resnet34, by approximately 0.77%. Additionally, DSCA-PSPNet records a recall of 93.21% and an F1 score of 92.38%. These metrics respectively highlight DSCA-PSPNet’s competence in accurately identifying true positives and maintaining a balanced performance between precision and recall.

Shifting focus to computational efficiency and resource consumption in **Table 3**, DSCA-PSPNet continues to shine. Although its prediction time of 4.57 ms for a single 512 × 512 image on RTX 3090 GPU is slightly slower than PSPnet-resnet34, it outperforms Unet, DeeplabV3+ and FPN considerably. Importantly, with 22.57M parameters, DSCA-PSPNet’s model complexity is on par with other models, showcasing that superior performance does not necessitate excessive complexity. Further, DSCA-PSPNet’s GFLOPs and memory usage affirm its efficiency, making it apt for deployment in resource-constrained scenarios.

**Table 3:** Accuracy metrics comparison for different segmentation methods.

Methods	IoU	Accuracy (%)	Precision (%)	Recall (%)	F1-Score
Unet (resnet34)	78.44	88.65	84.69	91.90	87.06
DeepLabV3+(resnet34)	81.83	90.62	86.65	92.40	90.31
FPN (resnet34)	79.84	89.79	93.13	84.88	88.59
PSPnet (resnet34)	83.18	92.25	91.64	91.52	89.49
DSCA-PSPNet	**87.58**	**92.34**	**93.80**	**93.21**	**92.38**

Bold value is the highest value.

Underline value is the second highest value.

In conclusion, the comprehensive evaluation presented in this section, through both qualitative and quantitative perspectives, cements the superiority of DSCA-PSPNet in sugarcane field segmentation. Its consistent lead across a variety of performance metrics, the demonstrated visual prowess, and efficient resource utilization collectively mark DSCA-PSPNet as a promising tool in the domain of sugarcane field segmentation and beyond. This underscores the applicability and potential of DSCA-PSPNet for real-world implementation, thus appealing to the academic community and sugarcane practitioners alike.

### 3.2 Ablation study

The ablation study aims to examine the progression of performance improvements that our proposed DSCA-PSPNet offers, starting from the baseline PSPNet(resnet34), and its variants augmented with sSE and cSE mechanisms, and finally to DSCA-PSPNet. Using sSE and cSE in the same position as the D-scSE in the models, ensures an unbiased and consistent basis for comparison.

A valuable tool in our analysis is the use of attention maps, generated from the output of the final layer of the backbone. This layer, rich with high-level semantic information, provides a detailed visual guide to how different models prioritize areas within an image.

**Figure 10:**
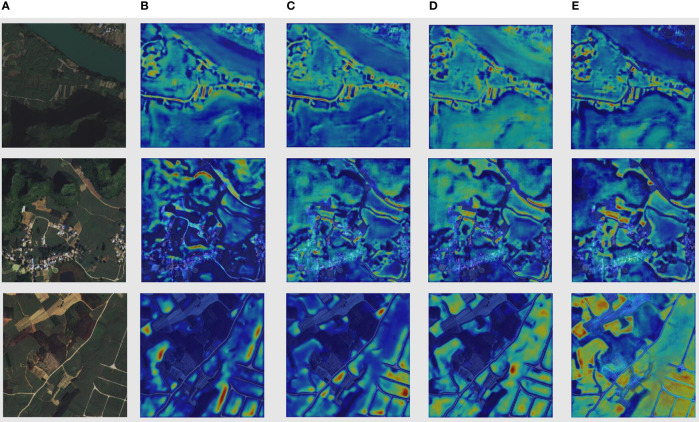
Columnnn **(A)** Original images. Columnn **(B)** Attention map of PSPnet. Columnn **(C)** Attention map of PSPnet+sSE. **(D)** Attention map of PSPnet+cSE. **(E)** Attention map of DSCAPSPNet.

The attention maps in **Figure 10**, column (A) presents the original images, and columns (B) to (E) show the attention maps for PSPNet, PSPNet+sSE, PSPNet+cSE, and DSCA-PSPNet, respectively. The difference in focus and detail becomes quite evident upon comparison. The baseline PSPNet exhibits less distinct segmentation, while the addition of sSE and cSE mechanisms enhances the model’s ability to distinguish different landforms more clearly. Yet, it is with DSCA-PSPNet that we observe the most significant concentration of attention on intricate agricultural details, such as edges and sugarcane fields. This confirms the superior capability of our D-scSE mechanism in capturing both local and global contextual details, enhancing the model’s understanding of the image.

Along with visual observations from attention maps, we perform a quantitative analysis on key performance metrics for each model variant, as represented in the tables below:

**Tables 4** and **5** shows that DSCA-PSPNet surpasses PSPnet and its sSE and cSE variants in all performance metrics. For example, in terms of IoU, DSCA-PSPNet outperforms the next best model, PSPnet+cSE, by 2.4 percentage points. This pattern continues with Accuracy%(2.09 percentage points higher), Precision% (0.16 percentage points higher), Recall% (1.69 percentage points higher), and F1-Score % (0.89 percentage points higher). These results confirm the effectiveness of the D-scSE module in improving DSCA-PSPNet’s performance.

**Table 4:** Performance metrics comparison for different segmentation methods.

Methods	Prediction Time (ms)	Parameters (Million)	GFLOPs	Memory Size (MB)
Unet(resnet34)	6.97	24.44	31.36	93.21
DeepLabV3+(resnet34)	5.98	22.44	31.62	85.60
FPN (resnet34)	7.24	23.16	27.49	88.33
PSPnet (resnet34)	**3.98**	**21.44**	**9.41**	**81.78**
DSCA-PSPNet	4.57	22.57	11.41	84.47

Bold value is the highest value.

Underline value is the second highest value.

**Table 5:** Accuracy metrics comparison in ablation study.

Methods	IoU	Accuracy (%)	Precision (%)	Recall (%)	F1-Score
PSPnet(resnet34)	83.18	92.25	91.64	91.52	89.49
PSPnet+sSE	84.76	92.79	92.13	92.88	90.59
PSPnet+cSE	85.18	93.25	93.64	91.52	91.49
DSCA-PSPNet	**87.58**	**92.34**	**93.80**	**93.21**	**92.38**

Bold value is the highest value.

In summary, our ablation study systematically evaluates the performance improvements of DSCA-PSPNet, beginning with the baseline PSPNet (ResNet34) and progressing through its variants augmented with sSE and cSE mechanisms, to the final DSCA-PSPNet model. This study not only quantitatively demonstrates DSCA-PSPNet’s superiority over its predecessors but also qualitatively underlines the effectiveness of our design choices, particularly the inclusion of the D-scSE module. By analyzing attention maps generated from the model’s final layer, we observed a significantly enhanced focus on critical sugarcane field details, such as field edges and textures, in DSCA-PSPNet compared to the baseline and other variants. Quantitative analysis reveals that DSCA-PSPNet surpasses other models in key performance metrics, including IoU, accuracy, precision, recall, and F1-score, confirming the D-scSE module’s pivotal role in improving segmentation capabilities. These results collectively highlight the D-scSE module’s contribution to the model’s overall efficacy in accurately segmenting complex sugarcane cultivation scenes, thereby validating the module’s integration as a critical enhancement in our deep learning architecture for precision agriculture applications.

## 4 Discussion

The primary limitation of the DSCA-PSPNet study is its reliance on a dataset exclusively from Guangxi’s Fusui County, captured on a single date. This limitation, while providing high accuracy within its narrow scope, raises concerns about the model’s robustness and adaptability to different sugarcane cultivation environments. The challenges in acquiring diverse, high-resolution satellite data, often restricted due to censorship and stringent data-sharing policies, combined with the intensive requirements of accurately labeling such imagery, have led to a lack of dataset diversity (Sing et al., 2021). Consequently, the model’s current iteration, although advanced, might not fully account for the variances in sugarcane fields across different geographical locations with varying environmental conditions and agricultural practices. A critical aspect yet to be verified is the model’s ability to accurately segment sugarcane fields in different stages of growth, under varying weather conditions, or in regions with distinct soil types (Lin et al., 2009). Addressing these challenges is imperative for future research. Efforts will be concentrated on expanding the model’s application to a broader range of sugarcane-producing regions worldwide. For instance, testing DSCA-PSPNet in countries like Brazil and India, which are major sugarcane producers but have different climatic conditions and cultivation practices compared to southern China and south east Asia, would be crucial. This would help assess the model’s adaptability and performance in diverse sugarcane farming contexts. Additionally, the examination of the model’s performance using multi-temporal satellite imagery is essential. This would offer insights into its capability to consistently recognize sugarcane fields throughout different growth stages and under varying seasonal weather patterns, such as the monsoon impact in South Asia or the dry season in Brazil. Collaborations with international agricultural research institutes, satellite imagery providers, and experts in global sugarcane cultivation could facilitate access to a more varied range of data, overcoming the limitations in data acquisition and labeling. Such collaborative efforts are vital in refining DSCA-PSPNet to address the unique challenges of sugarcane field segmentation in different parts of the world. Enhancing the model’s accuracy and versatility in this manner is not only crucial for advancing precision agriculture in the context of sugarcane farming but also has broader implications for sustainable agricultural practices and food security globally.

## 5 Conclusion

In the pursuit of sustainable agricultural practices, precise and accurate crop field segmentation remains a critical concern. Addressing this need, this study introduces the DSCA-PSPNet, a deep learning model specifically designed for sugarcane field segmentation. The integration of a modified ResNet34 backbone with PSPNet and D-scSE blocks is pivotal to the model’s success. The modified ResNet34 backbone, enhanced with dilated blocks, serves as a robust foundation for feature extraction, capitalizing on its deep residual learning framework to circumvent issues like vanishing gradients in deeper networks. These dilated blocks significantly augment the network’s capability for feature extraction, enabling the model to cover a wider field of view, thus capturing more contextual information without compromising resolution or incurring additional computational costs (Zhang and Zhang, 2021). The PSPNet component further assists in aggregating contextual information across various scales, crucial for differentiating sugarcane fields from other similar features in satellite imagery. The D-scSE blocks add a dynamic aspect to the model by recalibrating the channel-wise and spatial features in the network, fine-tuning the focus on relevant features for precise segmentation. Together, these elements enable DSCA-PSPNet to effectively navigate the spectral and spatial complexities inherent in agricultural landscapes. This design has enabled the model to achieve an IoU of 87.58%, an accuracy of 92.34%, a precision of 93.8%, a recall of 93.21%, and an F1-Score of 92.38%. These figures demonstrate its superior performance over established models. Moreover, DSCA-PSPNet proves to be computationally efficient, with a memory size of 84.47MB and a model size of 22.57MB.

In addition to developing the model, this study has compiled a comprehensive high-resolution satellite imagery dataset from Guangxi’s Fusui County, encompassing a broad spectrum of environmental conditions and field characteristics. This dataset provides a challenging yet realistic testing ground for DSCA-PSPNet, contributing significantly to the validation and refinement of the model. Furthermore, it represents a valuable resource for future research and innovation in the field of agricultural segmentation. The insights gained from this study not only demonstrate the potential of DSCA-PSPNet in sugarcane field segmentation but also highlight the model’s adaptability and potential applicability to other crop types. Future research could leverage this model and dataset to explore segmentation in different agricultural contexts, potentially expanding the scope of precision agriculture. By integrating these advances with ongoing research efforts, there is a strong potential for models like DSCA-PSPNet to play a pivotal role in enhancing sustainable farming practices, thereby contributing significantly to global food security and sustainable development goals.

## Data availability statement

The datasets presented in this study can be found in online repositories. The names of the repository/repositories and accession number(s) can be found below: https://github.com/JulioYuan/DSCA-PSPNet/tree/main.

## Author contributions

YY: Conceptualization, Methodology, Validation, Visualization, Writing – original draft. LY: Formal Analysis, Funding acquisition, Project administration, Resources, Supervision, Writing – review & editing. KC: Formal Analysis, Methodology, Writing – review & editing. YH: Data curation, Resources, Validation, Writing – review & editing. HY: Investigation, Project administration, Writing – review & editing. JW: Data curation, Formal Analysis, Methodology, Writing – review & editing.
